# Treatment of Gravitational Pulling Sensation in Patients With Mal de Debarquement Syndrome (MdDS): A Model-Based Approach

**DOI:** 10.3389/fnint.2022.801817

**Published:** 2022-05-23

**Authors:** Sergei B. Yakushin, Theodore Raphan, Catherine Cho

**Affiliations:** ^1^Department of Neurology, Icahn School of Medicine at Mount Sinai, New York, NY, United States; ^2^Institute for Neural and Intelligent Systems, Department of Computer and Information Science, Brooklyn College of the City University of New York, Brooklyn, NY, United States; ^3^Department of Computer Science, Graduate Center of CUNY, New York, NY, United States; ^4^Ph.D Program in Psychology and Neuroscience, Graduate Center of CUNY, New York, NY, United States; ^5^Department Neurology and Otolaryngology, NYU Robert I. Grossman School of Medicine, New York, NY, United States

**Keywords:** MdDS, velocity storage, orientation-vector, gravitational pull, rocking, swaying, bobbing, vestibular only (VO) neurons

## Abstract

Perception of the spatial vertical is important for maintaining and stabilizing vertical posture during body motion. The velocity storage pathway of vestibulo-ocular reflex (VOR), which integrates vestibular, optokinetic, and proprioception in the vestibular nuclei vestibular-only (VO) neurons, has spatio-temporal properties that are defined by eigenvalues and eigenvectors of its system matrix. The yaw, pitch and roll eigenvectors are normally aligned with the spatial vertical and corresponding head axes. Misalignment of the roll eigenvector with the head axes was hypothesized to be an important contributor to the oscillating vertigo during MdDS. Based on this, a treatment protocol was developed using simultaneous horizontal opto-kinetic stimulation and head roll (OKS-VOR). This protocol was not effective in alleviating the MdDS pulling sensations. A model was developed, which shows how maladaptation of the yaw eigenvector relative to the head yaw, either forward, back, or side down, could be responsible for the pulling sensation that subjects experience. The model predicted the sometimes counter-intuitive OKS directions that would be most effective in re-adapting the yaw eigenvector to alleviate the pulling sensation in MdDS. Model predictions were consistent with the treatment of 50 patients with a gravitational pulling sensation as the dominant feature. Overall, pulling symptoms in 72% of patients were immediately alleviated after the treatment and lasted for 3 years after the treatment in 58% of patients. The treatment also alleviated the pulling sensation in patients where pulling was not the dominant feature. Thus, the OKS method has a long-lasting effect comparable to that of OKS-VOR readaptation. The study elucidates how the spatio-temporal organization of velocity storage stabilizes upright posture and how maladaptation of the yaw eigenvector generates MdDS pulling sensations. Thus, this study introduces a new way to treat gravitational pull which could be used alone or in combination with previously proposed VOR readaptation techniques.

## Introduction

Mal de Debarquement Syndrome (MdDS) is a debilitating neurological condition characterized by non-spinning vertigo akin to being on a boat (Brown and Baloh, 1987; Cha, 2015). Patients have described the symptoms as rocking, swaying, bobbing, walking on a trampoline or walking on sponges (non-spinning vertigo), which are often associated with anxiety, depression, and cognitive issues (Hain et al., 1999; Cha et al., 2008). In a large number of instances, there is also a gravitational pulling sensation (Dai et al., 2017). MdDS symptoms are generally triggered by boat, plane, or car rides and have been referred to as motion-triggered MdDS (MT). A phenotypically similar disorder includes these symptoms, but the onset is due to non-motion triggers or comes on spontaneously. This has been referred to as “non-motion triggered - motion oscillating vertigo by the International Consensus of Vestibular Disorders (Cha et al., 2020). For our purpose, we will refer to this entity as non-MT MdDS”. MdDS is differentiated from other vestibular disorders because there is symptom relief when re-exposed to passive motion. Non-spinning vertigo persists while sitting, standing, and lying down long after the triggering event. Patients also report a sensation of gravitational pulling in one or several directions (Dai et al., 2017; Yakushin et al., 2020). These sensations were commonly accompanied by sensitivity to moving visual stimuli, loud noise, and fluorescent lighting, ear fullness, head pressure, brain fog, fatigue, and sensitivity to head movement. Patients often had cognitive complaints such as an inability to multitask, impaired concentration, and slower speech (Dai et al., 2017; Cha et al., 2020). The perceptions of motion (rocking and swaying) have been hypothesized to represent centrally induced maladaptation of the spatiotemporal coordination present in normal subjects (Dai et al., 2014; Cohen et al., 2018). When the non-spinning vertigo improved by readaptation, the cognitive symptoms immediately improved (Dai et al., 2017). However, the underlying deficiencies in pulling sensation, have not been addressed, and an effective treatment protocol has not been developed.

In the past, MdDS patients had up to 19 but on average 2–5 visits to physicians before being accurately diagnosed (Macke et al., 2012; Mucci et al., 2018a). The accuracy of an MdDS diagnosis depends on the awareness of the medical and research community of this syndrome. Lately, due to the internet, many patients are self-diagnosed and then confirm their condition with a specialist familiar with MdDS (Dai et al., 2017). The options for MdDS are therefor limited. Vestibular physical vestibular rehabilitation, benzodiazepines, and migraine medications can improve the quality of life in some patients, but symptoms remain in many others (Cha, 2012; Hain and Cherchi, 2016; Ghavami et al., 2017; Cha et al., 2018). The disruption of the inappropriate activity in a neural functional-connectivity network using non-invasive brain stimulation methods during several days may reduce symptoms (Cha et al., 2016, 2019; Ahn et al., 2021); however, the long-term outcome of this treatment is unknown.

Dai et al. (2014) proposed an effective therapy of MdDS based on the readaptation of the functional component of the vestibulo-ocular reflex (VOR) called velocity storage. The treatment concept was based on several experiments investigating the spatial orientation of velocity storage in the monkey (Raphan and Cohen, 2002; Cohen and Raphan, 2004). It was hypothesized that roll velocity storage eigenvector had become misaligned with the head roll axis during exposure to complex vestibular and visual stimuli, triggering inappropriate nystagmus and oscillating vertigo. The protocol developed was designed to counteract the roll eigenvector maladaptation by activating velocity storage in the direction opposite to what induced the contextual maladaptation. This readaptation is proposed to be induced by the OKS while rolling the head at the frequency of the patient’s oscillatory vertigo (OKS-VOR) (Dai et al., 2014). Roll readaptation treatment was effective initially in 75% MT and in 50% non-MT MdDS patients (Dai et al., 2017). A 1-year follow-up of these patients determined that the success rate for both MT and non-MT MdDS was identical at 50% (Dai et al., 2017).

A weakness in the OKS-VOR treatment was the lack of a placebo arm, although the effectiveness of this readaptation treatment has been independently confirmed by several investigators (Hain, 2018; Mucci et al., 2018b; Schenk et al., 2018). Furthermore, Mucci and colleagues (Mucci et al., 2018b) demonstrated that OKS-VOR readaptation is more effective than placebo. The original studies by Dai et al. (2014, 2017), as well as replications using this protocol (Hain, 2018; Mucci et al., 2018b; Schenk et al., 2018), demonstrated similar success rates regardless of slight variations in treatment setups and protocols. A subset of cases that initially worsened or did not respond to the treatment, later improved by reversing OKS stimulus, supporting Dai’s hypothesis that countering velocity storage should improve symptoms and that readaptation in the direction of velocity storage should increase MdDS symptoms (Schenk et al., 2018; Yakushin et al., 2020). This strengthened the argument that the results of Dai’s studies were not due to placebo and that velocity storage was at the root of the various syndromes associated with MdDS.

Since 2014, 591 MT and non-MT MdDS patients were treated at Icahn School of Medicine at Mount Sinai with the original VOR readaptation method (Dai et al., 2017). Among them were 50 patients treated only with a modified method of treatment for the gravitational pulling sensation. These 50 patients are the core of this study. The sensation of gravitational pulling, however, did not seem to improve with the original OKS-VOR readaptation protocol (Dai et al., 2014; Yakushin et al., 2020). We, therefore, worked on modifying the original treatment based on the idea that the yaw and pitch eigenvectors could have become maladapted. To accomplish this goal, this study retrospectively examined data from patients whose dominant debilitating syndrome was the gravitational pull sensation and was treated with OKS in a specific direction keeping the patient’s head stationary upright. An extension of the velocity storage model was then considered to explain how proprioception affects velocity storage. The main questions that we wished to answer was whether this model predicted how the gravitational pulling sensation might be induced by a maladaptation of spatial and temporal coding of the yaw axis eigenvector of velocity storage. We also wished to determine if the model predicted the orientation of OKS stimuli that would optimally re-adapt the eigenvector to alleviate the symptoms of the pulling sensation. Finally, we wished to determine whether the model predictions agreed favorably with the data on pulling directions and whether the alleviation of pulling sensations were alleviated by OKS, independent of whether the pulling sensation was a dominant feature of the debilitation or was only a component embedded in other features of MdDS.

## Materials and Methods

### Patient Selection

All MdDS patients seeking treatment with the VOR readaptation protocol at MSSM were screened with an intake form and a brief interview. Patient eligibility criteria were the same as for the subjects who were recruited in our previous studies (Dai et al., 2014, 2017; Yakushin et al., 2020). The inclusion criteria were (1) continuous rocking, swaying, bobbing, and/or gravitational pulling, which had persisted for at least 3 weeks, with or without a trigger. (2) Subjects reporting improvement in symptoms when in a moving vehicle (i.e., a car) and return of symptoms when stopped (Cha et al., 2020). (3) No history of head or neck trauma, Lyme disease, serious peripheral vestibular disease, or other major neurological disorders. There was no age limitation in this study. The patients were categorized as MT MdDS if the oscillating sensation began less than 2 days after a motion event and as non-MT MdDS if a motion event did not precede the onset of oscillating sensation or it took place more than 2 days after a motion event. Subjects were referred by physicians, physiotherapists, former patients, or were self-referred. Many had completed neurologic and otologic workups, including MRIs that were unremarkable. Institutional Review Board (IRB) approved a review of the records at Icahn School of Medicine at Mount Sinai under the grant listed in the acknowledgments.

### The Treatment Procedure for Gravitational Pull

**FIGURE 1:**
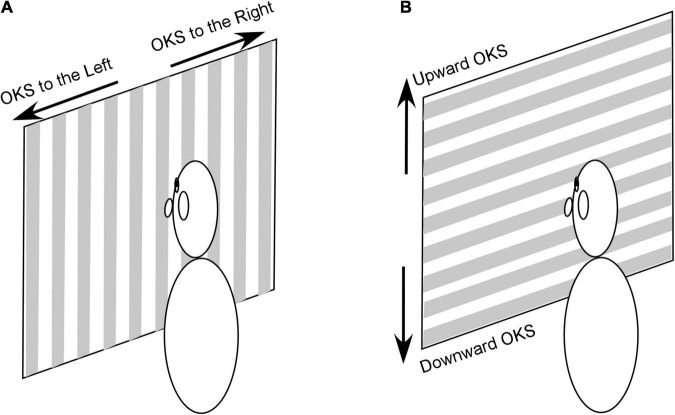
OKS stimulation used to induce horizontal **(A)** and vertical **(B)** optokinetic nystagmus. It should be noted that the chamber wall is cylindric and stripes in **(A,B)** are curved and wider at the periphery. See text for a detailed description of the stimulus.

Among 591 treated patients, 50 experienced a dominant sensation of gravitational pull. They were therefore treated with OKS to alleviate this symptom. Patients were seated upright in an enclosed cylindrical chamber. Horizontal optokinetic nystagmus was induced by rotating the projector about the vertical axis. The projector was located above the patient’s chair (Dai et al., 2014, 2017). The thickness of the stripes was 70 mm for the projected light and 110 mm for the shadows (Figure 1A). The projector could be tilted by 90°, to induce vertical optokinetic nystagmus (Figure 1B). Because the chamber was cylindrical, the horizontally projected stripes were slightly curved, and the widths of white and gray stripes varied 80–120 mm and 110–140 mm, respectively. Based on our previous study (Dai et al., 2017) a velocity of 5°/s and a brightness of 2 lux were used. The OKS stripes were administered for 1 min, and patients were required to stare at a point on the wall.

After each OKS, the patient was asked whether the postural stability improved, remained the same, or worsened. If the patient reported improvement or no change, the treatment time was increased. If the patient reported worsening of pulling, the direction of the treatment was reversed and given for 1 min. If subjects reported alleviation of symptoms, the velocity could be increased to 10°/s, and brightness increased to 3 lux as tolerated. A treatment session of 5–60 min was provided once a day.

### Quantitative and Qualitative Analyses of the Treatment Effectiveness

Subjective quantitative evaluation of improvement was evaluated with an eleven-point numerical Likert-like scale, where 0 represented “no MdDS symptoms” and 10 represented the severest symptoms the patient could imagine (Likert, 1932). The treatment was continued until the overall subjective symptoms score was improved by 50% or until no further improvements were reported. Thus, an average treatment for gravitational pull sensation over 4–5 days was 30 min, varying from 1 to 178 min. After each OKS exposure, patients were asked whether the symptoms improved, worsened, or had no effect. This qualitative response was used as a guideline for the next OKS treatment.

### Static Posturography

Static posturography for stability was performed using a Wii board (Dai et al., 2017). The dominant oscillating frequency of rocking or swaying was determined from the power spectra of the recorded center of pressure (COP) (Demura et al., 2008). Posture was recorded in several positions: feet 27 cm apart with eyes open, feet apart with eyes closed, feet together with eyes closed. Prior to 2017, to express an internal sensation of motion, subjects were asked to move their arm attached to an acceleration sensor at the frequency of the internally sensed movement (Dai et al., 2014). From 2017 onward, subjects were asked to move their bodies in the direction of perceived movement to exaggerate postural shifts while standing on a Wii board instead of using the arm. Postural data for comparisons were collected from recordings that were not exaggerated on the first day prior to the treatment and after the last treatment. Data were also collected at other times if subjects had difficulty determining the type of motion experienced and whether symptoms were improving or worsening. Again, these data were from recordings that were not exaggerated.

We define symmetrical body oscillation about the upright position in the for-aft plane (pitch) as rocking and side-to-side (roll) as swaying. Patients with gravitational pull sensations typically had asymmetrical body motions between upright and direction of pull. They also perceived their body motion, like oscillations in the plane of pull combined with resistance to pull.

To compare the postural stability after individual treatments, the displacement of COP over a 20-s period was computed as well as the root mean square (RMS) of the postural displacement along roll RMS, swaying, and pitch axes (pitch RMS, rocking) (Dai et al., 2017). Since body motion for the majority of patients in this study was not symmetrical and sinusoidal, the trace duration over 20 s was considered as the most reliable measure.

### Long Term Follow-Up

To determine the long-term effects of the treatment, all former patients were contacted first with a follow-up announcement letter. All patients who did not wish to participate in follow-up were excluded. Furthermore, all patients that were diagnosed with severe neurological problems since the treatment were also excluded. Among 50 patients with only gravitational pull sensation, one refused to participate in the follow-up. The follow-up forms include overall and individual symptom scores immediately after treatment, 2 weeks, and 1, 3, 6, and 12 months after treatment, as well as scores at the time of the follow-up (varied among patients). Follow-up forms also included VVAS, SVQ, DHI, BAI, and STAI to evaluate sensitivity to visual (VVAS) and physical (SVQ) motions, disability level (DHI), and the level of anxiety (BAI, STAI). To normalize individual physical, emotional and functional disability with DHI, which varies with the number of elements in each sub-scale, we used average individual scores which vary from 0 to 3.

### Other Relevant Data

Among the remaining 541 patients that experienced rocking and swaying, gravitational pull was reported by 376 patients (59 male). Because exposure to OKS can potentially trigger migraine-like symptoms (Dai et al., 2017), the treatment protocol for MdDS patients was designed to reduce major symptoms of MdDS within the shortest amount of time. We targeted the next most prominent symptom if the most bothersome symptoms (rocking, swaying, gravitational pull) resolved or resulted in no improvement. Thus, the original VOR-OKS readaptation protocol in these patients was combined with treatment for gravitational pull. To verify whether the treatment of gravitational pull was effective in these patients, we analyzed whether patients felt qualitative changes in symptoms. The long-term follow-up was not performed on these patients because it was not possible to determine whether OKS-VOR readaptation or OKS treatment was more effective in overall symptoms improvements in these patients.

### Statistical Analyses

Two groups of data were compared with a standard Chi-Square test. Multiple groups of data were compared by ANOVA using the Bonferroni *post hoc* test (Keppel, 1991). Sinusoidal fit through the data was performed using a least-square fit algorithm. Mean and standard deviations were presented as **a(b)**.

## Results

### The Theoretical Basis for the Onset of Mal de Debarquement Syndrome and Its Treatment

**FIGURE 2:**
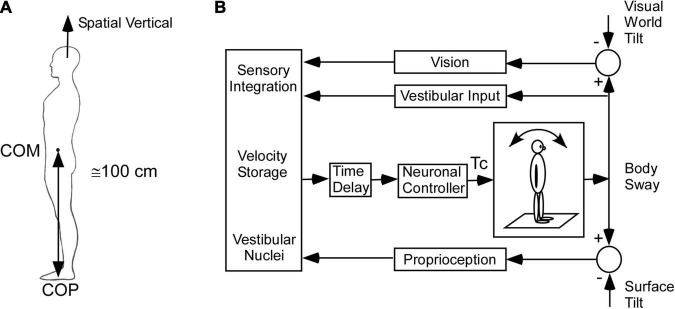
Model of the postural control. **(A)** Inverted pendulum model. COM – center of mass, COP – center of pressure. **(B)** Model of sensory integration of visual, vestibular, and proprioceptive inputs [modified from Horak (2009)]. This model includes the low frequency postural adjustments contributed by velocity storage.

Under normal circumstances, head and body posture is maintained so that the yaw axis of the head is aligned with the spatial vertical, which is opposite to that of gravity (Figure 2A; Horak, 2009; Ivanenko and Gurfinkel, 2018). Because the body center of mass (COM) is about 100 cm above the center of pressure (COP) at the foot and has a small base of support, it behaves as an inverted pendulum when standing and thus represents an unstable equilibrium (Nashner et al., 1989; Horak, 2009). It is, therefore, necessary for the central nervous system (CNS) to combine sensory information from the visual system, vestibular system, and proprioception to activate a neural controller to minimize body sway and maintain equilibrium (Figure 2B).

Much work has been done to characterize the neural control for maintenance of upright body posture by tilting the surface of the support and visual world tilt as part of a feedback control system and studying the effects on body sway (see Horak, 2009; Ivanenko and Gurfinkel, 2018 for review). A number of studies have included pulling the body for-aft as another important input, which can perturb upright stance and requires multisensory control to stabilize upright posture (Peterka, 2002, 2003; Mergner et al., 2003; Maurer and Peterka, 2005; Cnyrim et al., 2009). The aim of the control models was to focus on the multisensory control that contributes to postural stability and predict the changes in postural control when there is vestibular loss compared to normal (Mergner and Rosemeier, 1998; Mergner and Glasauer, 1999; Mergner et al., 2003; Maurer and Peterka, 2005). However, this kind of control accentuates the upper frequency (0.05–0.4 Hz) “rapid postural responses” that maintain upright posture and equilibrium and is not consistent with the slow oscillations and pulling characteristic of MdDS. Neither, do these models consider how the spatio-temporal or orientation properties of velocity storage maintain upright postural stability or how their maladaptation contributes to MdDS.

**FIGURE 3:**
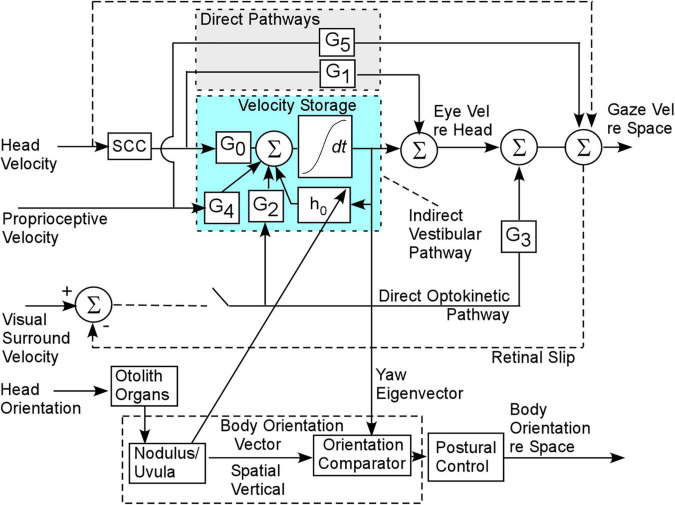
Gaze and body postural control contributed by the velocity storage integrator. G_0_ is the gain coupling matrix from the semicircular canals (SCC) to velocity storage. G_2_ is the gain matrix coupling the optokinetic velocity input to velocity storage. G_4_ is the gain coupling matrix from the proprioceptive velocity input to velocity storage. G_1_ and G_3_ are the direct pathway gain matrices from the semicircular canals and optokinetic input and are responsible for the rapid responses to head and optokinetic movement. There is also a rapid proprioceptive pathway (G_5_), but these are assumed not to play a role in the maladaptation leading to MdDS.

**FIGURE 4:**
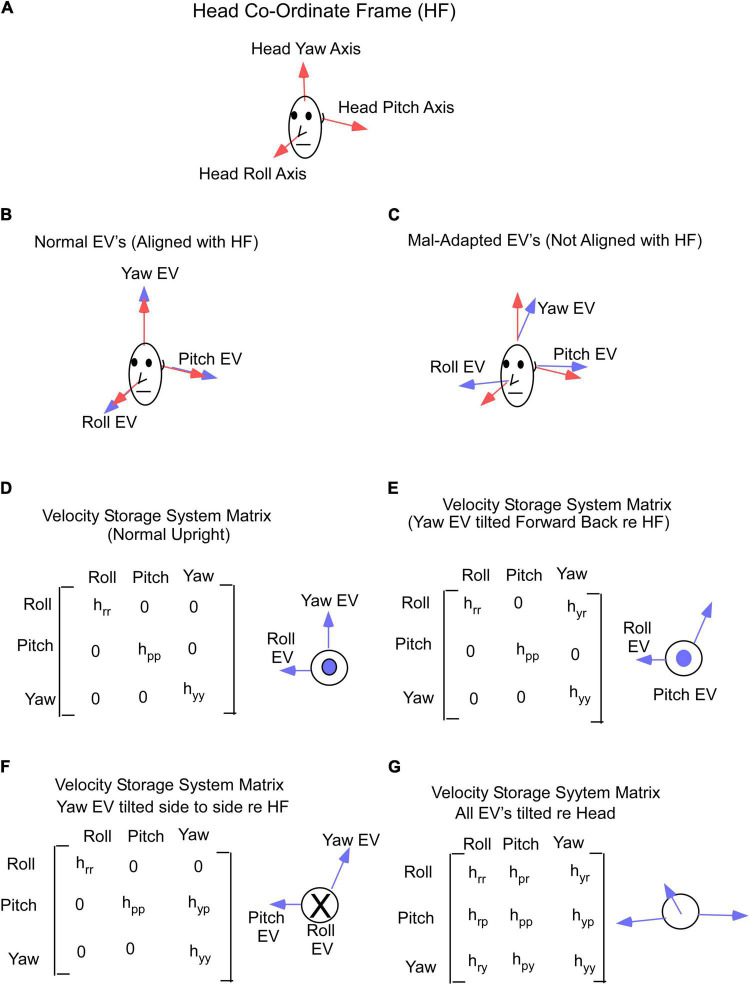
**(A)** Head coordinate frame used in this study. **(B)** Yaw, pitch and roll eigenvectors in normal non-adapted states and are aligned with the head axes. **(C)** Yaw, pitch and roll eigenvectors shifted from their normal orthogonal orientations due to maladaptation of the velocity storage system matrix. **(D)** Gain matrix of velocity storage in normal state, when yaw, pitch, and roll eigenvectors are aligned with the head axes. **(E,F)** Gain matrix of velocity storage with yaw eigenvector shifted forward **(E)** or sideways **(F)**. **(G)** Gain matrix of velocity storage when all 3 eigenvectors are shifted from their normal **(D)** orientations. Insets in **(D–G)** on the right from gain matrices are a top view of the head. EV – eigenvector.

The basis of our model and treatment protocol for MdDS comes from the hypothesis that the cause of the MdDS disorder is a disruption of the velocity storage mechanism, which initially was shown to store information about Head Velocity relative to space in the central vestibular system (Cohen et al., 1977; Raphan et al., 1979; Figure 3). It was subsequently shown that velocity storage has strong input from proprioceptive mechanisms that maintain posture during locomotion (Solomon and Cohen, 1992a,b; Figure 3, Proprioceptive Velocity). Velocity storage of motion information has orientation properties that are related to positions of the head relative to gravity (Sturm and Raphan, 1988; Dai et al., 1991; Figure 4) and its maladaptation could induce the slow postural adjustments readjustments. Normally, the head coordinate frame (Figure 4A, blue arrows) has the eigenvectors of H aligned with the head frame (Figure 4B, red arrows), with the H matrix being diagonal (Figure 4D). When the H matrix is non-diagonal, having a h_*yr*_ component, the Yaw eigenvector is pitched forward or back (Figure 4E). When the H matrix has a h_*yp*_ component, the eigenvector is rolled to the side (Figure 4F). In general, the H matrix can have all components, inducing a non-orthogonal basis for velocity orientation, which are not aligned with the head roll, pitch, and yaw axes (Figure 4G). Moreover, the orientation vectors that characterize velocity storage can be adapted by conflicting motion environments (Dai et al., 2009). Thus, velocity storage is a critical mechanism for converting central velocity coding and orientation from the vestibular system and inducing slow compensatory ocular and the postural response of the orientation vectors of velocity storage during long-term vestibular stimulation on sea voyages.

### Determining the OKS Direction to Treat Gravitational Pull

**FIGURE 5:**
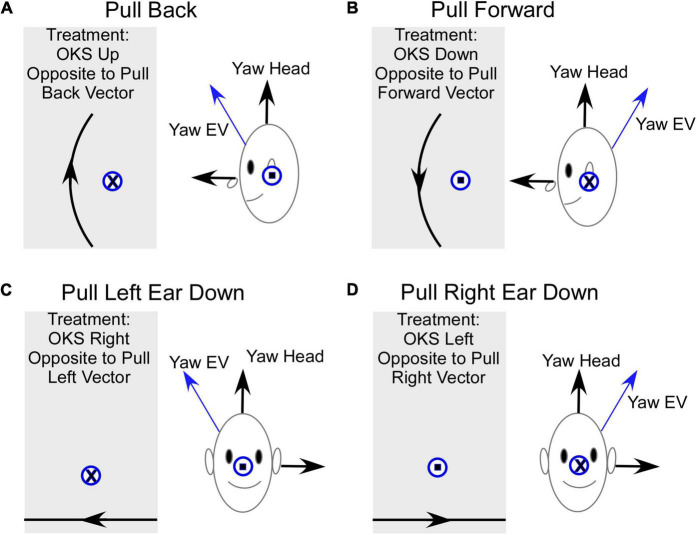
Summary of the model-based predicted treatment protocol for gravitational pulling sensation. EV- Eigenvector. **(A)** Sideview of the pulling back sensation. The Yaw EV is shifted forward so the head yaw is back relative to the EV. The vector representing this pulling back sensation is the cross product of Yaw Head with Yaw EV and according to a right-hand rule, this is a vector out of the left ear (circle with a dot). The OKS treatment is OKS up, which re-adapts the Yaw EV toward Yaw Head. The direction of rotation of OKS represented by a circle with an x, opposite to the maladapted vector rotation. **(B)** Sideview of the pulling forward sensation. The Yaw EV is maladapted back re the Yaw Head, inducing a forward pulling sensation. The vector for this rotation is into the left ear (circle with an x) and the OKS stimulus to readapt is down and represented by a vector out of the left ear. **(C)** Pull Left ear down. This occurs when the Yaw EV is rotated right. The appropriate treatment using horizontal OKS is toward the right, which because of the known cross-coupling of velocity storage would adapt the Yaw EV toward the Yaw Head. It should be noted that the model predicts that a more potent OKS stimulus to re-adapt this shift in the EV would be a counterclockwise roll OKS from the subject viewpoint, which would be a vector opposing the rotation of the Yaw EV relative to the Yaw Head. **(D)** Pull Right Ear down. This occurs when the Yaw EV is rotated left relative to the Yaw Head. The appropriate treatment using horizontal OKS is toward the left, which because of the known cross-coupling of velocity storage would adapt the Yaw EV toward the Yaw Head. In this instance, the model predicts that a more potent OKS stimulus to re-adapt this shift in the EV would be a clockwise roll OKS from the subject viewpoint, which would be a vector opposing the rotation of the Yaw EV relative to the Yaw Head. Front view. Gray rectangles – screens with OKS stimulus. The direction of OKS is indicated by arrows. The direction of the OKS vectors for side down pulling sensation is consistent with previously studied cross-coupling when the head is tilted side down.

The model suggests that the mechanism of treatment for the gravitational pulling sensation is the adaptation of the yaw axis eigenvector of velocity storage (Figure 5) to align with the yaw axis of the head. When the yaw axis eigenvector maladapts to having a positive roll component (counterclockwise rotation), then the yaw axis of the head is tilted back relative to the yaw eigenvector (Figure 5A). We hypothesize that there is a cross-product computation of the head yaw vector with the yaw eigenvector, which, using the right-hand rule, encodes a vector coming out of the left ear, which is an orientation back (Figure 5A, blue circle with dot). This creates an internal sensation of pulling back. The treatment for adapting the yaw eigenvector so that it aligns with the head would be an upward OKS stimulus, which opposes the maladapted orientation using the right-hand rule (Figure 5A, a gray area, circle with x). This is counter-intuitive to the notion that a downward OKS is necessary for a backward pulling sensation. Similarly, a maladapted tilt back of the Yaw eigenvector (Figure 5B) induces an orientation back and a pull forward sensation as the head yaw axis is down relative to the yaw eigenvector (Figure 5B, a circle with x). Again, the treatment would be a counter-intuitive OKS down, which would produce a vector opposite to the orientation of the pull forward vector according to the right-hand rule (Figure 5B, a gray area, a circle with dot).

A somewhat similar approach can be taken to explain the treatment for pulling to the right or left. If the yaw eigenvector maladapts by tilting to the right, then the yaw axis of the head is rotated left ear down relative to the eigenvector, giving a pull to the left side (Figure 5C). To cancel out the vertical component of the yaw eigenvector, the OKS stimulus should be opposite to the pull. This should be OKS to the right against the gravitational pull (Figure 5C; Raphan and Cohen, 1988). When the yaw eigenvector is maladapted by rotation counterclockwise, the pull is right ear down (Figure 5D). To cancel out the vertical component of the eigenvector, the OKS adaptation should be OKS to the left (Figure 5D). We predict from the direction of the maladaptation that roll (torsional) OKS stimuli, which would oppose the pull orientation, would perhaps be more efficient in the treatment of the lateral pulls. In addition, the model has not considered how alterations of the eigenvectors of velocity storage affect perception, which has been considered during off-vertical axis rotation and its effects on motion sickness (Dai et al., 2010). The incorporation of motion sickness into this model and how it is controlled by perception would clarify some of these issues.

We further assumed that magnitude of the vector could also play a role in MdDS symptoms. To account for up and down pulling, we hypothesize that this is caused by a maladaptation of the magnitude of the yaw eigenvector. A reduction in the magnitude would induce a floating sensation, while an increase in magnitude would induce heaviness. The appropriate adaptation is upward OKS for the floating sensation to increase the magnitude of the yaw eigenvector and downward OKS for the heaviness to decrease the magnitude of the eigenvector.

### Treatment of Patients With Gravitational Pull Sensation as the Dominant Symptom

**FIGURE 6:**
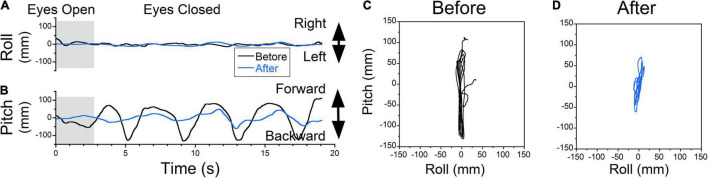
The COP changes as a function of time (Static posturography). **(A)** Side-to-side (swaying) body oscillations. **(B)** Forward-back (rocking) body oscillations. Static posturography of a 52-year-old female with MdDS triggered immediately after a flight. Prior to treatment, the patient had no swaying (**A**, black trace) but reported strong backward gravitational pulling (**B**, black trace). The shaded areas in **(A,B)** are intervals when the patient had her eyes open. **(C,D)** Trajectory plots of COP before **(C)** and after **(D)** exposure of upward OKS at 5°/s for 1 min. After treatment, postural stability increased (**A,B,D** blue traces).

**FIGURE 7:**
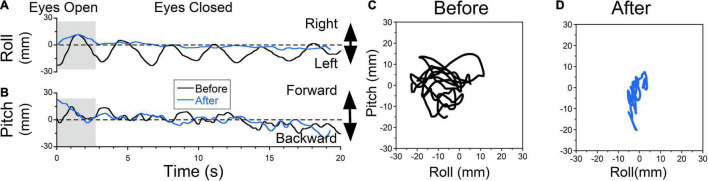
Static posturography of a 48-year-old female with MdDS triggered immediately after a cruise. Details of individual plots are described in Figure 6. The patient experienced a sensation of gravitational pulling to the left (**A**, black trace) and some rocking (**B**, black trace). When the eyes were closed, the patient’s body gradually drifted to the left. When the posture reached a certain deviation from upright, to gain stability patient quickly moved her body back to the upright position. This is different from body swaying when oscillation is sinusoidal and body deviations are symmetrical about upright. After exposure to OKS to the right at 5°/s for 2 min, the sensation of gravitational pulling to the left was reduced (**C**, blue traces). As a result, side-to-side body oscillations were no longer observed. Minimal forward-back body oscillations remained unchanged (**D**, blue traces). **(D)** Trajectory plots of COP before **(C)** and after **(D)** treatment. After treatment, postural stability increased (**A,B,D** blue traces).

**FIGURE 8:**
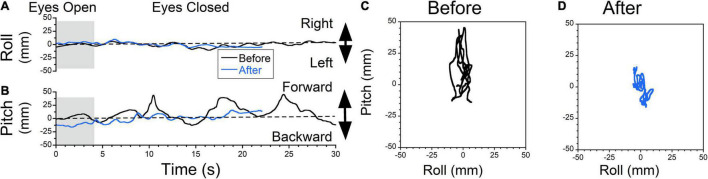
Static posturography of a 71-year-old female with MdDS triggered immediately after a cruise. Details of individual plots are described in Figure 6. The patient reported a sensation of forward gravitational pulling. Body deviation side-to-side were minimal (**A**, black traces). When the patient closed her eyes, the body was gradually falling forward, but when deviation reached certain level, it was pulled back toward upright position (**B**, black trace). After exposure to downward OKS at 5°/s for 2 min, the swaying remained minimal (**C**, blue trace) and forward body deviation was reduced **(D)**. Self-score of overall symptoms prior to treatment was 7/10, and after treatment was 4.5/10.

Based on the above model predictions, we compared the use of upward OKS to treat gravitational pulling back and up, downward OKS to treat pulling forward and downward, leftward OKS to treat pulling right, and rightward OKS to treat pulling left. Of the 50 patients with dominant symptoms of gravitational pull sensation, 41 were females (82%). The age of the MdDS patients with dominant pulling sensation was 49 (12) for females and 31 (15) for males (*p* = 0007). MT onset of MdDS symptoms was reported by 33 patients and 17 had non-MT MdDS symptoms. There was no difference in the average duration of MdDS from the onset time until the time of treatment for both groups 3.2 (6.2) years, varying from 3 weeks to 30 years. The most common triggers for MT MdDS were cruises (36%), boating (36%), flight (18%), and car rides (10%). Among non-MT MdDS, 6 patients associated the onset of MdDS with vertigo attacks (35%). Among the other triggers were brushing teeth, massages, and elevator rides (one of each). The majority (47%) were unable to identify the event associated with symptom onset. There was a high number of males in non-MT (35%) vs. MT MdDS (9%). The average subjective symptom scores based on the Likert scale prior to treatment did not vary between MT and non-MT MdDS and was 5.5 (2.3). Besides the gravitational pull sensation, patients also reported non-dominant sensations of rocking, swaying, bobbing, trampoline walking, and other symptoms commonly reported by patients with MdDS (Table 1). Immediate responses to treatment for gravitational pull sensations in different directions are shown in Figures 6–8.

**TABLE 1:** Symptoms reported by 50 MdDS patients with gravitational pull as the dominant sensation of motion.

Symptom	Occurrence
Rocking	62%
Swaying	78%
Bobbing	40%
Trampoline walking	52%
Migraine	28%
Tinnitus	36%
Brain fig	66%
Head pressure	44%
Fullness of the ears	44%
Anxiety	76%
Depression	54%
Fatigue	68%

### Posturography Analysis of Pulling Backward Sensation Before and After Treatment

The static posturography measurement of a patient who experienced a dominant gravitational pulling back sensation had minimal roll oscillations but substantial pitch oscillations with a dominant frequency of ≈0.27 Hz (Figure 6A, black trace). The pitching oscillations were not symmetrical, with larger amplitudes and faster pitching backward, which corresponded to a sensation of backward pulling that was resisted by the patient leaning forward (Figure 6B, black trace).

Postural stability improved after upward OKS at 5°/s for 1 min (Figures 6A,B, blue traces). Plots of roll vs. pitch COP show the phase relationship of body motion as well as the power of any pulling offset before and after treatment (Figures 6C,D). Before treatment, the power (RMS) and stability (Trace) of the upright posture were trace 20s = 2,061 mm, roll RMS = 5 mm, pitch RMS = 71 mm. After treatment, trace 20s = 347 mm, roll RMS = 4 mm, pitch RMS = 12 mm, which is an 83% improvement in stability (Trace). Patients also reported a subjective improvement in backward pulling.

### Posturography Analysis of Pulling Lateral Sensation Before and After Treatment

Treatment of a patient that experienced a sensation of gravitational pull to the left is shown in Figure 7 (Figure 7A, black trace). The patient constantly resisted the pull by bringing the body back to an upright position, which generated oscillations at ≈0.3 Hz. This was accompanied by small irregular roll oscillations (Figure 7B, black trace). On static posturography, it appeared that there were constant oscillations ≈ ± 15 mm in all directions (Figure 7C, trace 20s = 387 mm, roll RMS = 7 mm, pitch RMS = 5 mm). After rightward horizontal OKS at 5°/s for 2 min, leftward pulling was not present (Figure 7A, blue trace) and was not reported subjectively. Irregular rocking remained the same (Figure 7B, blue traces). Static posturography revealed a reduction of leftward pulling (Figure 7D, trace 20s = 189 mm, roll RMS = 1 mm, pitch RMS = 3 mm). The pitching oscillations were about the same (roll RMS = 5mm vs. 3 mm). The trace length was reduced by 51%.

### Posturography Analysis of Pulling Forward Sensation Before and After Treatment

The typical example of treatment for forward pulling is shown in Figure 8. The patient did not experience any swaying (Figure 8A, black trace), but reported forward pulling (Figure 8B, black trace). The patient resisted the pulling creating non-sinusoidal body oscillations ≈0.12 Hz. The static posturography confirmed this oscillation at ± 30 mm (Figure 8C, trace 20s = 310 mm, roll RMS = 3 mm, pitch RMS = 13 mm). The patient was exposed to downward OKS at 5°/s for 2 min. After this treatment, sway was minimal (Figure 8A, blue trace), and rocking was reduced (Figure 8B, blue trace). The static posturography confirmed this improvement (Figure 8D, trace 20s = 184 mm, roll RMS = 3 mm, pitch RMS = 5 mm). The trace length was improved by 41%.

### Treatment of Pull-Up and Pull-Down Sensation

The treatment effect for gravitational pull-up and down sensations could not be revealed with static posturography and was justified only qualitatively. Because the direction of gravitational pull could change on different days, data from the 50 subjects treated for various gravitational pulls were analyzed to determine the effectiveness of the treatment with sex and trigger as variables rather than comparing the effectiveness of the treatment of individual pull directions.

Treatment was initially successful in 36 (72%) patients (73% MT and 71% non-MT MdDS). Body motions were larger with eye-closed conditions and significantly reduced in successfully treated patients from 727 (682) mm to 406 (433) mm (44%, *p* = 0.03). The postural improvement with the eye-opened condition was insignificant (39%, *p* = 0.089). In patients who did not report significant symptoms improvement after the treatment, postural stability significantly improved with eye-opened [411 (230) vs. 234 (68), 43%, *p* = 0.017] but not with eye-closed (*p* = 0.940). This indicates that postural stability is not a major factor in the overall severity of MdDS as judged by patients.

Since resistance to gravitational pulls produces body oscillations similar to sinusoidal motion, we compared available data of the rocking and swaying frequencies of 47 patients who experienced dominant gravitational pull sensations with the sensations obtained from 541 MdDS patients where the pulling sensation was not dominant. The rocking frequency of patients with dominant gravitational pull sensations was comparable to that of other MdDS patients [0.27 (0.14) Hz vs. 0.25 (0.17) Hz, *p* = 0.568]. The same was true for the swaying of patients with the sensation of pull only [0.33 (0.19) Hz vs. 0.31 (0.19) Hz, *p* = 0.619]. Thus, the body oscillations of patients who experienced dominant gravitational pull sensations were similar to those of the patients with dominant rocking and swaying sensations.

### Follow-Up Study

Thirty-four of 49 patients treated for dominant gravitational pull responded to the follow-up questionnaire, with 16 providing only an overall symptoms score. Since patients were treated at different times, the follow-up was performed 2.8 (1.3) years after the treatment. There were 20 (20/34) patients who reported improvement, and 11 (11/34) reported no improvement at the time of follow-up. Thus, 59% (20/34) of patients treated only for gravitational pull remained improved 3 years later (54% MT, 57% SO). Two more MT patients (2/34) did not respond to the treatment, but about a year later, one started clonazepan, and another changed their diet, and by the time of the follow-up, symptoms were significantly improved. One other non-MT (1/34) patient was diagnosed with a small fiber neuropathy which caused severe pain and it could not be determined which symptoms were due to MdDS.

Among patients who reported improvements at follow-up, 15 (15/20) had improvement immediately after the treatment and remained improved. Five others (5/20) did not respond to the treatment immediately and, most likely, recovered spontaneously at the time of follow-up. Finally, among the patients who did not report improvement at the last follow-up, 8 (8/11) initially responded to the treatment, but symptoms returned after traveling home. Thus, we could conclude that all the patients that improved immediately and maintained improvement after travel home continued to have benefits up to 3 years later.

Individual symptoms were analyzed to determine whether long-term treatment effectiveness could be predicted based on the data obtained immediately after treatment (Table 2).

**TABLE 2:** Frequency of individual symptoms at long-term follow-up in successfully and unsuccessfully treated groups.

Symptoms	Successfully treated			Not successfully treated		
	Experienced	Score	Improve	Experienced	Score	Improve
Overall	100%	6.3 (2.2)	100%	100%	5.9 (2.5)	0%
Rocking	100%	7.2 (1.6)	100%	100%	6.1 (2.7)	27%
Gravity pulling	100%	6.9 (1.7)	100%	100%	5.6 (2.8)	64%
Brain fog	86%	5.1 (2.5)	83%	100%	4.1 (2.9)	50%
Light’s sensitivity	57%	3.4 (3.5)	75%	67%	3.7 (3.8)	50%
Noise sensitivity	71%	3.9 (3.2)	60%	56%	2.7 (2.8)	40%
Anxiety	83%	5.3 (3.1)	60%	100%	6.7 (2.5)	44%
Depression	83%	5.3 (3.7)	60%	83%	4.6 (3.8)	44%
Fatigue	83%	5.9 (4.3)	40%	89%	6.1 (3.2)	50%
Fuzzy vision	17%	0.2 (0.4)	100%	56%	2.2 (3.0)	40%
Head pressure	83%	4.3 (3.3)	60%	78%	2.7 (2.7)	57%
Ear fullness	50%	2.9 (3.8)	100%	56%	1.9 (3.2)	40%

*Experienced: present of patients who experience this symptom. Score: severity of that symptom on 0–10 self-score prior to treatment. Improve: percent of patients who reported at least 50% improvement of that symptom. Score is presented as mean (SD).*

Detailed analyses of individual symptoms score indicate that the was no difference in individual scores of successfully treated and not successfully treated groups (*p* > 0.127). The most commonly experienced symptoms were rocking, gravitational pull, and anxiety. It was not surprising that all patients also reported improvement in rocking since they perceived the gravitational pull sensation as fore-aft rocking. What was surprising was that 100% of successfully treated patients reported improvement in fuzzy vision and ear fullness, while only ≈55% in the unsuccessfully treated group improved. The second-largest improvement in successfully treated patients was the brain fog (83%) and sensitivity to fluorescent lights (75%). Improvement of all other symptoms was only slightly larger in the successfully treated group. Interestingly, while the overall score in the unsuccessfully treated group was not improved, the treatment improved the sensation of gravitational pull in 64% of the unsuccessfully treated group. Twenty-seven percent in that group also reported improvement of the rocking sensation. Thus, we can speculate that when postural improvements are associated with reduction of fuzzy vision, ear fullness, brain fog, and sensitivity to lights, overall improvement will remain. Data also indicated that when sensitivity to lights remained high, symptoms will be re-triggered after successful treatment of MdDS.

We performed quantitative analyses of sensitivities to visual stimuli (VVAS) and physical motion (SVQ) in 2 groups. Only 1/7 patients had high sensitivity to visual stimuli (VVAS = 3.8) in the successfully treated group, while the remaining 6 had low sensitivity (av VVAS = 1.5). In the non-successfully treated group, 1/10 had extreme sensitivity (VVAS = 8.7), three patients had high sensitivity (av VVAS = 4.5), and six patients had low sensitivity (av VVAS = 1.6). Sensitivity to the physical motion was moderate in two groups, with 71% successfully and 55% not-successfully treated patients remained sensitive to motion, while the remaining patients were not sensitive to motion. Regardless of differences in sensitivities to moving visual stimuli and to physical motion, patients with higher visual sensitivity were more likely to be sensitive to physical motion (MLR, *p* < 0.05). General disability was lower in the successfully treated group (DHI = 26.4 vs. 40.9). The same was true for physical (0.9 vs. 1.4), emotional (0.9 vs. 1.4), and functional (1.3 vs. 1.9). Thus, the physical and emotional disabilities remain high in the non-successfully treated group.

Anxiety level was verified by several different scales STAI Y1 and STAI Y2, BAI. STAI indicates moderate anxiety in both groups (45 of 80, where < 40 is normal). Anxiety was severe in the majority (86%) of successfully treated patients but mild in the majority (70%) of the unsuccessfully treated as determined by BAI. Similarly, the anxiety level was abnormal in 60% of successfully treated and only in 20% of unsuccessfully treated patients as determined by HADS(a). Thus, if the patient with high anxiety responded to treatment initially, the high anxiety was not a significant factor in long-lasting treatment success.

### Qualitative Analyses of Gravitational Pull Sensation Treatment in 591 Patients

Data from 50 patients with dominant sensation of gravitational pull and 376 patients that experience gravitational pull as one of several other motion sensations were combined (376 + 50 = 426). The most common direction of the gravitational pulling was backward (52%) and sideways (32%). Pulling forward (11%), down (5%), and up (3%) were less frequent. Thus, backward gravitational pulling was the most frequent, dominant sensation. During the 4-day treatment, some patients experienced a pulling sensation only in one specific direction, while other patients experienced pulling sensations for which the direction of pull varied over time.

A backward gravitational pulling sensation was reported by 307 patients (307/426, 72%). The backward only pulling sensation was exclusively experienced by 57%, whereas 35% experienced a backward pulling sensation and one other additional pulling direction, 5% experienced two other additional pulling directions, and 3% experienced three other additional pulling directions.

The majority of patients with gravitational pull-back sensation reported improvement of postural stability after exposure to upward OKS (96%, 296/307), similar to that shown in Figure 6. Six patients (6/307, 2%) did not report any postural changes after upward OKS. The five remaining (5/307, 2%) patients reported improvement after downward OKS but no improvement after upward OKS. None of 307 patients reported worsening of their symptoms after upward OKS. Thus, backward pulling was reduced after upward OKS in 96% of patients who experienced this sensation.

The mean treatment time for backward pulling was 17 (19) min, varying from 1 to 135 min over a week of treatment. There was no difference in treatment times for patients who experienced only back pull or pull in multiple directions (*p* = 0.420, ANOVA with Bonferroni adjustment).

Lateral pulling was reported by 190 patients (89 left, 101 right). Only 26% experienced pulling in one direction. The majority (63%) experienced pulling in two directions, while 9 and 2% experienced pulling in three and four directions, respectively. Thus, pulling in two directions was the most common sensation in patients who experienced lateral pulling. Data were found where lateral pulling, patients were exposed to OKS in the direction opposite to the sensation of pulling (Figure 5). The average treatment time was 15 (21) min for leftward pulling and 18 (25) min for rightward pulling. The results were combined because there was no difference in treatment duration (*t*-test, *p* = 0.502). The average treatment time for lateral pulling of 188 patients was 17 (23) min varying from 1 to 171 min over a week of treatment.

The treatment for lateral pulling was effective in 95% of patients (181/190). Seven patients (4%, 7/190) did not report any improvement. One patient reported improvement after OKS was induced in the same direction as pull (1/190), and another after OKS was induced in either direction (1/190).

Forward pulling was reported by 66 patients (66/426, 15%). Forty-eight percent reported pulling only in one direction, while 30% had it in two, 11% in three, and 11% in four directions. Thus, similar to gravitational pull backward, gravitational pulling forward was frequently the only direction of pull experienced by patients. The average treatment duration was 10 (12) min, varying from 1 to 65 min over a week of treatment.

The treatment for gravitational pull forward was effective in 94% (62/66) of patients. Two patients (3%, 2/66) reported improvement after upward and downward OKS, and 2 other patients (3%, 2/66) did not report significant improvement.

Upward pulling was reported by 18 patients (18/426, 4%). Patients frequently described this sensation as floating above the ground or not being grounded. Thirty-three percent of patients experienced isolated upward pulling. Many patients had additional pulling directions (28% in two, 28% in four, and 11% in three). The average treatment duration was 10 (13) min over a week of treatment. Seventeen patients (94%, 17/18) reported improvement after this treatment, and one (6%) reported no significant changes.

Downward pulling was reported by 33 patients (33/426, 8%). These patients frequently reported that their legs were heavy. Thirty-four percent reported isolated downward pulling. The majority (41%) reported additional pulling in one more direction. Pulling in 3 directions was reported by 19% and in 4 directions by 6% of patients. The average duration of treatment was 19 (33) min, varying from 1 min to 154 min over a week of treatment. Eighty-five percent (28/33) of patients reported improvement after downward OKS.

Thus, while the average duration of treatment for gravitational pulling in different directions varied from 10 to 17 min, this difference was not significant (*p* = 0.120, ANOVA). Pulling in only one direction was most common for backward pulling (57%) and forward pulling (48%) sensations. Pulling in all other directions was multidirectional.

### Effects of Pull Sensation Treatment in the Wrong Direction of OKS

The model predicted that the treatment of backward and forward pulling sensation with forward and backward OKS, respectively, would exacerbate the symptoms. We experimentally verified the effect of OKS in the wrong direction. We found data where downward OKS for 1 min was used to treat backward pull in 26 patients. Twenty of 26 (77%) reported worsening of the pull sensation. The remaining 6 of 26 did not report any changes. Thus, downward OKS was ineffective in treatment gravitational pull backward, confirming that treatment for yaw eigenvector correction is following the right-hand rule as predicted by the model (Figure 5).

Similarly, to test whether hypotheses derived direction of effective treatment is correct (Figure 5), 2 patients with lateral pull sensations were exposed to OKS in the same direction and reported symptoms worsening.

To test whether downward pulling could be treated with upward OKS, it was applied to 6 of 33 patients with that pulling sensation. In one patient, 1 min exposure to upward OKS increased the sensation of downward pulling. The other 5 (5/33) reported improvement after upward and downward OKS.

Thus, using short 1 min OKS in the direction which is opposite to that predicted by the model is typically worsening the sensation of pulling.

### Treatment of Oscillating vs. Pulling Vertigo

In some instances, patients failed to distinguish the difference between lateral pulling, swaying, forward/backward pulling, and rocking. In many cases, the gravitational pulling can be determined by posturography. Body rocking and swaying were typically sinusoidal at a specific frequency (Dai et al., 2014, 2017). Thus, posturography was helpful in identifying gravitational pulling when it revealed non-sinusoidal, somewhat nystagmus oscillations with varying frequencies, such as that shown in Figures 6–8. Moreover, rocking and swaying typically had equal amplitude in both directions from the upright center position. In the case of gravitational pulling, posturography typically revealed oscillations away and back to the upright center position (Figures 7A, 8B, dashed lines).

**FIGURE 9:**
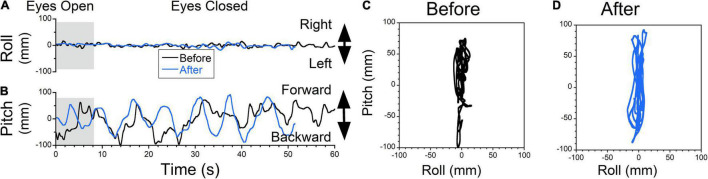
Static posturography of a 52-year-old female with MdDS of unknown origin (possibly swimming). Details of individual plots are described in Figure 6. The patient did not experience any swaying (**A**, black trace) but reported a sensation of rocking and backwards gravitational pulling (**B**, black trace). Forward-back motion was irregular but deviations forward and back were of similar amplitude. Thus, static posturography did not have a typical pattern of rocking or gravitational pull sensation forward or backward. After exposure to OKS to the right at 5°/s while rolling the head at 0.1 Hz for 3 min (treatment for rocking), swaying remained minimal (**A**, blue trace) but rocking became stronger and regular at 0.15 Hz (**D**, blue traces). The patient reported worsening of her symptoms. At the same time fore-aft oscillations became regular **(C,D)**. The appropriate treatment should have been upward OKS for the pulling back sensation. This shows that inappropriate treatment, based on some “intuitive” notion and not model-based could have deleterious consequences.

A more complicated case is shown in Figure 9. The patient did not experience any swaying (Figure 9A, gray trace) but had substantial fore = aft body rocking at ≈0.1 Hz (Figure 9B, gray trace). Oscillations were not very consistent but were symmetrical about the upright center position (trace 20s = 812 mm, roll RMS = 4 mm, pitch RMS = 27 mm) (Figure 9C). We first attempted to treat the patient for fore-aft rocking. Rightward OKN was induced at 5°/s, while the head was rolled side-to-side at 0.1 Hz for 3 min. Following treatment, the patient had no changes in swaying (Figure 9A, blue trace) but reported a stronger rocking sensation (Figure 9B, blue trace). Static posturography revealed oscillations at 0.15 Hz (trace 20s = 716 mm, roll RMS = 6 mm, pitch RMS = 54 mm). The roll RMS values were close to zero before and after that treatment. The pitch RMS, however, increased by 100%. Since trace duration was about the same in both cases, an increase in pitch RMS indicates that this treatment induced the fore-aft rocking. Based on this result, the treatment was reversed to eliminate induced rocking, and the forward pulling was successfully treated (not shown). This indicates that the original protocol using VOR-OKS readaptation (Dai et al., 2014) may not be appropriate for treating gravitational pull sensations.

## Discussion

This study has shown that the sensation of gravitational pulling experienced by MdDS was related to the maladaptation of the orientation of the yaw axis eigenvector of velocity storage. As such, patients could be treated by OKS in a specific direction determined by the direction of pulling as predicted by the model according to the right-hand rule (Figure 5). The pulling sensation could be alleviated regardless of whether it was the dominant symptom or was part of the symptoms experienced by MdDS patients whose dominant features were pitch or roll oscillations. Treatment was effective in 72% of patients immediately after the treatment, and symptoms remained improved 3 years after the treatment at 58% of patients. This indicates that OKS itself is a robust treatment for the gravitational pull and is further evidence that maladaptation of velocity storage, which is accessed by OKS is the root cause of MdDS.

The eigenvectors of velocity storage represent a central vestibular motion reference of space (Dai et al., 1991; Raphan and Sturm, 1991; Raphan and Cohen, 2002; Cohen and Raphan, 2004). Lengthy exposure to conflicting vestibular environments might induce “false” coding of space, especially the spatial vertical, which is defined by the acceleration of gravity (Raphan and Cohen, 2002; Cohen and Raphan, 2004). Based on this false coding, the eigenvectors of velocity storage adapt and become embedded as the representation of space (Dai et al., 2014). Thus, an internal mismatch of the orientation vectors of velocity storage, i.e., its eigenvectors, with that of the direction of the head axes induces a disequilibrium. This disequilibrium may cause the body to oscillate or experience a pulling sensation. The basis of the treatment described in this study is that the time constant of velocity storage and its eigenvectors can be adapted by countering conflicting visual-vestibular input, oscillations or pulling sensations induced by MdDS can be corrected. This forms the basis of the protocols tested for eliminating the symptoms of MdDS.

The original treatment of MdDS, was based on the idea that the roll eigenvector of velocity storage had maladapted toward pitch during cross-axis stimulation (Dai et al., 2009, Dai et al., 2014). Therefore, the protocol developed for treatment was to use a combined OKS and vestibular stimulus, which presumably re-aligned the eigenvectors with the head axes (Dai et al., 2017; Yakushin et al., 2020). This protocol, however, could not explain the sensation of pulling experienced by some MdDS patients (Dai et al., 2017; Yakushin et al., 2020) and was not effective in treating this symptom. In this study, we demonstrated that maladaptation of the yaw eigenvector alone predicts the direction of the pulling sensation by a misalignment of the yaw eigenvector with the head yaw axis and that an OKS stimulus that aligns the yaw eigenvector with the head yaw axis is effective in the treatment of pulling.

The model-based directions of the OKS that promote effective treatment are important because when the treatment is in the opposite direction, it may exacerbate the pulling sensation problem. For example, according to the model, a pull sensation backward is due to the yaw eigenvector being maladapted forward, causing a misalignment with the head yaw axis. A readaptation strategy should therefore be upward to re-align the yaw eigenvector toward the head yaw axis. This is seemingly “counter-intuitive,” since the OKS is in the same direction as the pulling. However, it is the yaw eigenvector that is being readapted toward the head yaw axis, which is the therapeutic direction. A downward OKS would exacerbate the pulling and cause more problems. A similar argument can be given for the downward pulling sensation. We have used horizontal OKS to treat side-down pulling sensation. This is effective because there is considerable cross-coupling from the yaw to roll, which corresponds to a side-down pulling sensation (Raphan and Cohen, 1988). However, other directions of OKS, such as roll OKS, could be more effective for side down pulling sensation, but this needs further study.

Another important aspect of this model-based study was that it showed that the OKS stimulus was effective in treating the pulling sensation regardless of whether it was the dominant feature of the MdDS or was embedded in rocking and swaying as the dominant features. As explained before, the gravitational pull sensation could also be mistakenly interpreted as oscillations (Figure 9). However, treatment for gravitational pull alone is different from OKS-VOR readaptation treatments of oscillatory vertigo. Furthermore, this study indicates that treatment of the gravitational pull by OKS with the head stationary does not cause significant symptoms to increase, while OKS combined with the head motion when OKS is in the wrong direction may significantly increase MdDS symptoms. Thus, when it is unclear whether the patient is experiencing the gravitational pulling or body oscillations, it is safe to test whether posture improves by first treating the gravitational pulling sensation. This suggests that patients in whom OKS-VOR readaptation is ineffective may benefit from OKS alone.

A model-based analysis of how specifically misalignment of the eigenvectors might cause the rocking and swaying in MdDS has not been developed. Our own studies and studies from the other laboratories indicate that 25% of patients with motion-triggered and 50% of patients with spontaneous onset of MdDS do not respond to OKS-VOR treatment (Dai et al., 2014, 2017; Hain, 2018; Mucci et al., 2018b). Furthermore, body side-to-side oscillations frequently experienced by MdDS patients (Dai et al., 2017), could be only explained by pitch eigenvector maladaptation as is proposed in the present study. This may indicate that while passive transportation is commonly affected by roll eigenvector orientation, possible maladaptation of yaw and pitch eigenvectors should also be considered. It further suggests that either yaw, pitch, or roll OKS treatment protocol may be effective in helping alleviate some symptoms because it has alleviated the pulling sensations. This goes along with an even lower success rate of spontaneous MdDS when either eigenvector has equal chances of becoming maladapted. Thus, developing a clearer model-based analysis of postural and eye movement dynamics may lead to improved treatment protocols for yaw and pitch eigenvector readaptation that may improve treatment outcomes.

How velocity storage is realized in three dimensions is not known. However, there is evidence that velocity storage integration comes about because of interconnections across the midline as well as connections among various types of vestibular-only (VO) neurons on each side (Cohen et al., 2018). The weights of the interconnections could form a large scale recurrent neural net (Raphan et al., 2019) that implements the system matrix and encodes the eigenvectors. Early experiments from our laboratory support this idea, showing that velocity storage and its spatial properties were coded by VO neurons (Reisine and Raphan, 1992; Yakushin et al., 2017). These VO neurons had been known to receive multiple convergent inputs from various semicircular canals and otoliths (Dickman and Angelaki, 2002; Yakushin et al., 2006; Eron et al., 2008b). We recently demonstrated that cross-coupling from horizontal to vertical and roll components of VOR were also coded by canal-otolith convergent VO neurons (Yakushin et al., 2017). Polarization vectors of VO neurons are flexible and tend to align their orientation with gravity (Eron et al., 2008a,^2018^), even when animals were in complete darkness without any specific training stimulus. This is distinct from polarization vectors coded by Eye-Head-Velocity (EHV) and Position-Vestibular-Pause (PVP) neurons that project to oculomotor neurons and therefore are part of the direct VOR pathway (Kolesnikova et al., 2011). There is also a distinct group of central otolith-only neurons that provide a rigid reference frame for head orientation (Schor et al., 1984, 1985; Angelaki et al., 1993). These neurons do not adapt their polarization vectors (Eron et al., 2009). Thus, the neural machinery exists that when the body is in the upright position a maladapted yaw eigenvector in MdDS patients can be misaligned with those neurons that encode the direction of gravity aligned with the yaw axis of the head.

The estimate of the direction of gravity in MdDS patients, however, largely relies on the polarization vector provided by VO neurons (the eigenvectors). The discrepancy between this estimate of gravity and the fixed co-ordinate frame provided by the otolith-only neurons could cause the sensation of gravitational pulling in that direction. A cross product, which gives the magnitude and direction of the misalignment of the vectors can also be implemented by another layer of the neural network, which gives the perception of pulling studied in this paper (Figure 4). We speculate that treatment with OKS has a strong corrective effect on the eigenvectors provided by VO neurons. As a result, coordinate frames provided by the two groups of neurons could be taught to align, which minimizes the sensation of gravitational pulling. In the present study, we demonstrated that exposing patients to full-field OKS that has a component whose vector is opposite to the cross product, corrects the gravitational pulling.

The majority of MdDS patients reported high sensitivity to the motion of their visual environment or to moving objects (visually induced dizziness, VID) (Dai et al., 2017; Mucci et al., 2018b). However, treatment of MdDS with readaptation of velocity storage (Dai et al., 2014, 2017) is based on patients’ exposure to a full field OKS, to which patients reported discomfort. Thus, treatment time was minimized to achieve a positive effect (Yakushin et al., 2020). Determining whether a patient is actually rocking or is pulling forward or backward with postural correction can minimize treatment time and side effects. The same is true in distinguishing the difference between the lateral pull and sway. Patients frequently fail to distinguish the difference between two sensations. Static posturography is also not always reliable in making the distinction between pulling and oscillating vertigo. Furthermore, while posturography seems to be a very attractive objective measure of MdDS, this study demonstrates that subjective severity of the overall MdDS symptoms which is accepted by most clinicians and researchers does not correlate with postural improvements.

How long does it take to induce improper learning, and why does that learning last so long? Previous studies of our laboratory indicate that when angular VOR is adapted in the context of gravity over 1 h, the contextual change can be observed for several days (Yakushin et al., 2003). Other laboratories have confirmed our findings and demonstrated that long-lasting changes of gravitational context could occur within several minutes (Schubert et al., 2008). Recent studies also indicate that otolith context plays a critical role in spatial perception, and 5 min of learning could significantly affect the perception of the spatial vertical axis (Tarnutzer et al., 2013, 2014). We speculate that MdDS is another example of long-lasting learning of a gravitational context. The correction back to normal did not occur spontaneously because it required exposure to the same context. This speculation is confirmed by several patients treated in our laboratory since 2014, who reported that another air flight or boat ride cured their symptoms. We further speculate that maladapted learning occurs on the level of the brainstem. Cortical areas are also involved since the majority of MdDS patients are suffering from anxiety, depression, fatigue, head pressure and headaches, cognitive impairment, and visual disturbance (Cha et al., 2012, 2021; Cha and Chakrapani, 2015). Involvement of the cortical areas may be due to brainstem input based on the neural pathways involved during full-field OKS exposure (Dai et al., 2017).

Though the above results are promising in improving the treatment of MdDS, there were several aspects of this study that need further work. First, there was no uniform treatment protocol for each patient. As mentioned above, the protocol was adjusted depending on the patient’s response. Although there was much variability, the predetermined direction of OKS based on symptoms was consistent for over > 90% of the patients with good results. Second, there was no placebo arm. However, a small subset of cases worsened when the stimulus was provided in the “wrong” direction, supporting the conclusion that the effect was more than placebo. Third, posturography, unfortunately, was not consistently performed on all patients, and subjective improvement was used as the outcome measurement. Lastly, the putative pathophysiology of MdDS in humans were based on animal studies that focused on the brainstem and cerebellum, and we cannot exclude concomitant cortical processes, which have been demonstrated in other studies (Cha and Chakrapani, 2015; Yuan et al., 2017; Cha et al., 2021). Despite these shortcomings, the study included a sound specific model as well a large number of patients with a significant response that agreed with model predictions.

Falling backwards, which could be related to a backward pulling sensation has been reported in groups of patients, including patients with cerebellar ataxia (van de Warrenburg et al., 2005). It is also well documented that backward falling is frequently reported by subjects with the bilateral vestibular loss (Ewald, 1892; Magnus, 1924). These conditions responded to treatment with upward and downward OKS (Vitte et al., 1994; Tsuzuku et al., 1995). We do not know whether OKS will provide any symptom relief in other groups of patients. However, this study clearly demonstrates that OKS alone has the potential to be a powerful tool in the correction of gravitational pulling in MdDS patients through a model-based analysis. If such treatment is useful in other diseases that manifest as gravitational pulling sensation, standardization of treatment is feasible. Further studies are required to determine the durability of the response and establish to what extent the treatment is reproducible. Thus, while there is more work to be done to develop the model and the model based analysis, the present study has established the foundation for how space and time is encoded in velocity storage through the eigenvectors and eigenvalues and how they might maladapt in diseased states. It also establishes a foundation for developing a sound protocol for alleviating pulling sensation symptoms, which do not exacerbate the MdDS problem.

## Data Availability Statement

The data analyzed in this study is subject to the following licenses/restrictions: The data are in a proprietary format (VMF) and are read only by a proprietary program written for this purpose. Requests to access these datasets should be directed to SY, sergei.yakushin@mssm.edu.

## Ethics Statement

The studies involving human participants were reviewed and approved by the IRB of the Mount Sinai School of Medicine. Written informed consent for participation was not required for this study in accordance with the national legislation and the institutional requirements.

## Author Contributions

SY contributed to the design of the experiments, MdDS treatments, data acquisition, and writing of the manuscript. TR contributed to the conceptual framework and data analysis that allowed for the comparison of the data to model predictions and to the organization and writing of the manuscript. CC contributed to the clinical evaluation of the patients as well as the writing of the manuscript. All authors approved the submitted version.

## Conflict of Interest

The authors declare that the research was conducted in the absence of any commercial or financial relationships that could be construed as a potential conflict of interest.

## Publisher’s Note

All claims expressed in this article are solely those of the authors and do not necessarily represent those of their affiliated organizations, or those of the publisher, the editors and the reviewers. Any product that may be evaluated in this article, or claim that may be made by its manufacturer, is not guaranteed or endorsed by the publisher.
